# Assessing the variation of DNA damage response of individual human cells exposed to photon radiation

**DOI:** 10.1007/s00411-026-01232-9

**Published:** 2026-06-16

**Authors:** Magdalena Płódowska, Dominika Drzazga, Aneta Węgierek-Ciuk, Przemysław Szyc, Beata Brzozowska, Paweł Wołowiec, Prabodha Kumar Meher, Halina Lisowska, Andrzej Wojcik

**Affiliations:** 1 Department of Medical Biology, Institute of Biology, Jan Kochanowski University, Kielce, Poland; 2 Biomedical Physics Division, Institute of Experimental Physics, University of Warsaw, Warsaw, Poland; 3 Department of Medical Physics, Holy Cross Cancer Centre, Kielce, Poland; 4 Centre for Radiation Protection Research, Department of Molecular Biosciences, the Wenner-Gren Institute, Stockholm University, Svante Arrhenius väg 20C 106 91 Stockholm, Stockholm, Sweden

**Keywords:** DNA damage, DNA repair, Single cell variability, Ionising radiation

## Abstract

Individual cells of the same type and in the same position in the cell cycle show stochastic cell-to-cell variability (noise) in gene and protein expression, even under the same environmental conditions. The aim of the study was to test if radiation-induced DNA damage response of cells shows noise, on top of the randomly distributed DNA damage. This was tested by comparing the frequency of DNA repair foci in sister nuclei of binucleated cells (intra BNC variability) and nuclei of two BNC (inter BNC variability). Inter cell variability was also measured for mononucleated cells (MNC). Binucleation was induced in U2OS cells by cytochalasin B. Cells were exposed to 2 Gy of photons, 53BP1 and γH2AX foci were visualised and counted in nuclei of binucleated and mononucleated cells 30, 60, 120 and 180 min post exposure. Coefficients of variation were calculated as indicators of variability. For both types of foci, the frequencies observed in sister nuclei within BNC were positively correlated and exhibited significantly lower variation compared to the variability measured between single nuclei from randomly paired BNC. The contribution of noise to the total variability was 28% for 53BP1 foci and 50% for γH2AX foci. The results demonstrate that the variability of focus frequency observed in nuclei of BNC results from a combination of noise and random damage variability.

**Supplementary Information:**

The online version contains supplementary material available at 10.1007/s00411-026-01232-9.

## Introduction

Intrinsic radiosensitivity can be defined as the inherent or inborn sensitivity of cells, tissues, or organisms to radiation, independent of external factors or the presence of effect modifiers. It is believed to be governed by factors that determine a cell’s ability to repair DNA damage and respond to radiation-induced stress (Szumiel 2008).

When synchronized cells from one cell line, which are expected to have a uniform intrinsic radiosensitivity, are irradiated under identical exposure conditions, the response to radiation is always diverse. For example, following exposure of HeLa cells in the late G1 phase to 2 Gy of X-rays results in death of 35% of cells (Tohyama et al. 2022). Exposure of human peripheral blood mononuclear cells (PBMC) in the G0 phase to 2 Gy of X-rays results in 41% cells showing one or more chromosomal aberrations (Edwards et al. 1979). The diversity is believed to result from the random distribution of ionisations inside cell nuclei (Lessler 1964), an assumption that is substantiated by the Poisson distribution of radiation-induced chromosomal aberrations (Edwards et al. 1979) and of γH2AX foci (Horn et al. 2011). If the diversity is solely a function of random distribution of ionisations, then it should be reproducible under reproducible exposure conditions.

It is now clear that individual cells of the same type and at the same state show marked cell-to-cell variability in gene expression, even under the same environmental conditions (Raj and van Oudenaarden 2008). The variability is only weakly related to a physiological state such as position in the cell cycle (Eberwine and Kim 2015). The stochastic expression of genes leads to a cell-to-cell variability, or noise (Thattai and van Oudenaarden 2001; Soltani et al. 2016) in protein copy numbers. This, in turn, leads to a random variability in cellular response to stress, including DNA damage repair (Uphoff and Kapanidis 2014) and death induced by lethal drugs (le Roux et al. 1998; Inde et al. 2020). Consequently, when cells are exposed to radiation, they should demonstrate diverse levels of momentary DNA damage response that are independent of the randomly variable amounts of deposited energy. Because DNA damage response (DDR) has an impact on the consequence of DNA damage, the momentary level of DDR can be assumed to reflect the momentary radiosensitivity. It would be interesting to separately quantify the level of variability of momentary radiosensitivity (defined here as noise) and the level of variability of radiation-induced level of damage (defined as random damage). Theoretically, if a cell would be exposed anew twice at the same moment in time, its reaction to radiation (intracellular variability) should be more similar than the reaction of two randomly selected cells (intercellular variability) irradiated at the same moment in time. Intracellular variability would reflect the random damage, while the intercellular variability would reflect the sum of random damage and noise (total variability). Thus, the relative contribution of noise to the total variability could be derived from the ratio of the intracellular variability to intercellular variability, subtracted from one. The present investigation was undertaken to test this hypothesis.

The major target for radiation effects of a cell is the DNA inside its nucleus (Szumiel 2008). Thus, measurement of the reaction of a cell that was exposed anew twice at the same moment in time is possible in cells that have two nuclei. Generating cells with two nuclei is possible with the help of cytochalasin B (Fenech and Morley 1986). To test the above-mentioned hypothesis, we irradiated binucleated U2OS cells with photons and compared 53BP1 and γH2AX foci in their sister nuclei reflecting the intracellular variability. The intercellular variability was assessed by comparing focus frequencies in single nuclei of two randomly selected binucleated cells. For comparison with binucleated cells, focus frequencies in nuclei of mononucleated cells were also analysed.

The reason for selecting U2OS was that these cells have a wild type p53 and express the green fluorescent protein (GFP)-tagged repair protein 53BP1 (Bekker-Jensen et al. 2005). We have successfully used these cells to study factors influencing DNA damage response to radiation (Plodowska et al. 2022, 2025). Here, the advantage of their use was in the possibility of measuring the variability of DDR at two levels: the accumulation of 53BP1 protein and phosphorylation of H2AX at sites of DSB repair. Limitations resulting from their use are described in the discussion.

## Materials and methods

### Cells and irradiation

**Fig. 1:**
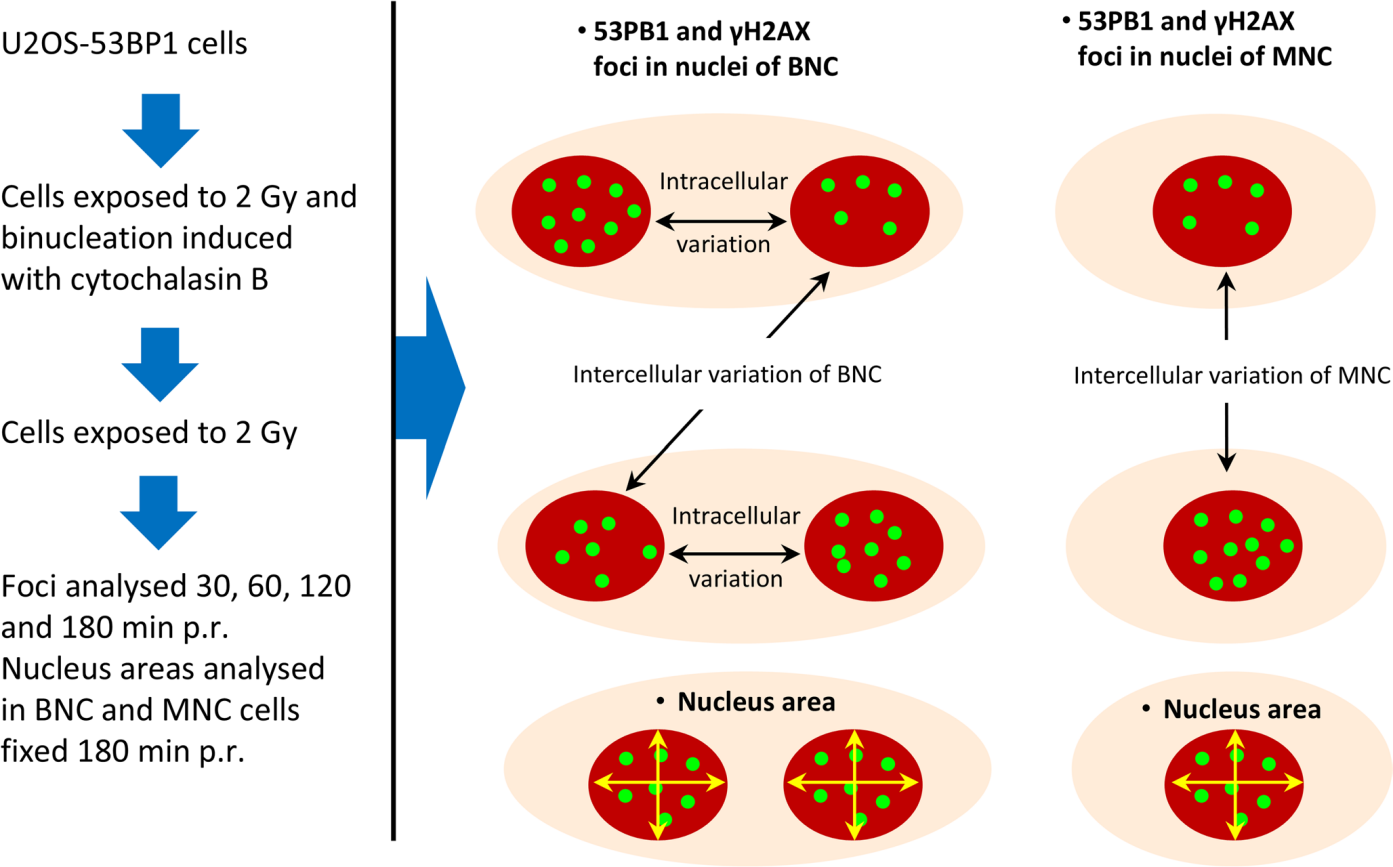
Scheme of the experimental setup. Intracellular variation of focus frequency in BNC was analysed by comparing the focus frequencies in sister nuclei of one BNC. Intercellular variation of focus frequency in BNC was analysed by comparing the focus frequencies in randomly selected single nuclei of two randomly selected BNC. BNC: binucleated cell; MNC: mononucleated cell; p.r.: post radiationem

A scheme of the experiments is shown in Fig. 1. Human osteosarcoma cells, U2OS, expressing a green fluorescent protein GFP-tagged repair protein 53BP1, were constructed as described elsewhere (Bekker-Jensen et al. 2005) and were kindly provided by Dr. C. Lukas. The cells were cultured at Jan Kochanowski University in Dulbecco modified Eagles medium (CORNING, cat. 10-014-CVR) supplemented with 10% bovine calf serum (Biowest, Fetal Bovine Serum Premium, cat. S181BH-500) and 1% penicillin streptomycin (Sigma-Aldrich) in a 5% CO_2_ humidified 37 °C incubator. At regular intervals cells were cultured in the presence of 400 µg/ml Geneticin-G418 (Sigma-Aldrich) to positively select cells expressing 53BP1-GFP. Before irradiation, cells were seeded on round glass coverslips that were placed in 6 well dishes.

Irradiation at room temperature (20 °C ) was carried out with 6 MeV photons using a medical LINAC (linear accelerator, Versa HD, Elekta, Sweden) at a dose rate of 1 Gy/min and a source-to-cell distance of 1 m. The dose was 2 Gy. Electron equilibrium was achieved by placing the 6 well dishes on top of a polymethyl methacrylate plate. Control cells were kept outside the LINAC bunker. The LINAC was operated at the Holy Cross Oncology Centre in Kielce, ca. 5 km away from the Jan Kochanowski university where the experiments were performed. Cells were transported between both locations by car inside a Styrofoam box. The transport time was less than 30 min. 3 independent experiments were carried out for analysis of 53BP1 foci and 3 additional experiments – for the analysis of γH2AX foci.

### Induction of binucleation and 53BP1 and γH2AX focus analysis

Our initial plan was to focus on 53BP1 foci and to include their analysis in micronuclei. Therefore, in order to induce micronuclei, U2OS cells were exposed to 2 Gy of radiation as described above and Cytochalasin B (Sigma-Aldrich) was added at a final concentration of 5 mg/ml for 24 h of incubation time to induce binucleation. Thereafter, cells were irradiated again with 2 Gy and fixed 30, 60, 120 and 180 min later for analysis of DNA repair foci. Foci in control cells were analysed together with cells fixed 30 min after exposure. The repair time started immediately after radiation exposure and included the transport in the Styrofoam box at room temperature. Because very few micronuclei were detected, focus analysis was only carried out in nuclei of binucleated cells (BNC) and of mononucleated cells (MNC).

53BP1 foci were visualised directly thanks to GFP tagging. γH2AX foci were detected by immunostaining. No counterstaining of nuclei was applied, as described previously (Plodowska et al. 2022). For 53BP1 focus analysis, cells on coverslips were fixed with 3% formaldehyde for 15 min at room temperature, washed with PBS and placed in 6-well plates filled with PBS for temporal storage. For analysis, a cover slip with cells was invertedly placed on a microscopic cavity slide with the well filled with 100 µl PBS. For γH2AX foci, cells on coverslips were fixed in ice-cold 90% methanol for 5 min, permeabilized in blocking buffer (skimmed milk + bovine serum albumin (BSA/Sigma-Aldrich) and incubated with anti-γH2AX antibody Ser139 (Upstate/Millipore) followed by the secondary anti-mouse antibody Alexa Fluor 546 (Invitrogen/Thermo Fisher). Both antibodies were diluted in PBS containing 2% bovine serum albumin (BSA). Cover slips were then placed in 6-well plates filled with PBS for temporal storage. For analysis, a cover slip with cells was invertedly placed on a microscopic cavity slide with the well filled with 100 µl PBS.

Fluorescence images were acquired using a Nikon A1 + laser-scanning confocal microscope (Nikon Instruments, Tokyo, Japan) equipped with a Plan Apo 40x DIC MN2 optics. For each field, a single optical section (one z-plane) was captured using identical microscope settings (laser power, detector gain, pinhole and scan speed 1/32) for all samples within each experiment. Images were saved as 1024 × 1024 pixel, 24-bit JPEG files and subsequently examined manually on a computer screen for focus counting and analysis. Because the analysis was based on manual counting of discrete foci rather than pixel intensity measurements, JPEG compression was deemed not to affect the scoring procedure.

Foci were enumerated manually by visual inspection of fluorescence images displayed on a computer monitor. Every distinct fluorescent focus recognizable as a signal was included in the analysis, regardless of its apparent size, morphology, or partial overlap with neighbouring foci. All image scoring was conducted in a blinded manner with respect to the treatment group and fixation time. At least 30 BNC and 30 MNC per fixation time point and experiment were analysed for 53BP1 foci by manually counting foci on images. The number of cells scored for γH2AX foci was lower because, following the analysis of 53BP1 foci, it was decided to check how far the results were reflected at the level of γH2AX foci. To this end, three additional experiments were carried out and the attempted number of cells analysed for γH2AX foci was 30 for the sum of all repeats per fixation time point. The number was reached for MNC at all fixation times and at for BNC at all fixation points except 60 and 180 min, where only 22 cells could be analysed per time point.

### Analysis of cell nucleus areas

The areas of nuclei in BNC and MNC of control cells (0 Gy) and of cells exposed to 2 Gy and harvested at 180 min post exposure were calculated by multiplying the major (longest) and minor (shortest) diameters of a nucleus (Fig. 1). Measurements were performed using a ruler on images with a constant resolution and magnification. The numbers of measured nuclei for control cells were: 60 and 20 nuclei for 53BP1 and γH2AX MNC images, respectively; 60 and 60 nuclei for 53BP1 and γH2AX BNC images. The numbers of measured nuclei for irradiated cells were: 90 and 122 nuclei for 53BP1 and γH2AX MNC images, respectively; 180 and 132 nuclei for 53BP1 and γH2AX BNC images. The measurement results are given in arbitrary units (a.u.).

### Monte Carlo calculation of ionisations in cell nuclei

The expected distribution of ionisations inside cell nuclei was calculated by Monte Carlo using the Geant4-DNA tool (https://geant4-dna.in2p3.fr/index.html), version 11.2.1. The simulation geometry consisted of a photon source, a cylindrical plexiglass phantom and cells placed on its surface. The phantom had a radius of 1000 μm and a thickness of 50 μm. The phantom thickness was chosen for visualization purposes, ensuring a comparable scale of ionisation events in both the phantom and the cells. A total of 100 binucleated and 100 mononucleated cells were randomly positioned on the phantom surface without spatial overlap. For simulation purposes, both the cytoplasm and the nuclei were modelled as water, assuming equivalent density and elemental composition.

BNCs were represented as ellipsoids with semi-axes of 30 × 50 × 30 μm, while MNCs were modelled as ellipsoids with semi-axes of 30 × 40 × 30 μm. The nuclei of both cell types were modelled as spheres with a radius of 15 μm. A monoenergetic 6 MeV photon beam, characterized by a uniform spatial distribution, was directed perpendicularly to the phantom surface. The beam cross-section was circular with a radius of 1000 μm, defined at a plane located 1 cm above the surface of the phantom. The total photon count was set to 10^8 (fluence of 3.18 × 10^13 m⁻²), ensuring a statistically significant number of ionisation events. The presented results originate from a single simulation run.

The simulation output included the spatial coordinates of individual ionisation events, enabling analysis of their distribution and correlations between the number of ionisations occurring within the nuclei of binucleated cells.

### Statistical analyses and calculating noise intensity

In order to compare the formation and decay of foci in BNC and MNC, mean focus frequencies at 0, 30, 60 120 and 180 min post exposure were fitted to a Gaussian model and areas under the curve (AUC) were calculated using SigmaPlot 16 (Grafiti LLC, Palo Alto, USA).

Coefficients of variation (CV) were calculated for focus frequencies between two nuclei of BNC (intra BNC variation), between two nuclei of two randomly selected BNC (inter BNC variation) and between nuclei of two randomly selected MNC (inter MNC variation). CV at 30, 60, 120 and 180 min post exposure between intra BNC and inter BNC and between BNC and MNC were compared by one way ANOVA with Holmak-Sidak test to account for multiple comparisons, using SigmaStat 4.0 (Systat Software Inc., USA). CV in control cells were not included in the comparative analyses. CV at 180 min post exposure for foci normalized to nucleus area between intra BNC and inter BNC and between BNC and MNC were compared by Kruskal-Wallis One Way Analysis of Variance on Ranks, using SigmaStat 4.0 (Systat Software Inc., USA).

The relative contribution of noise (N) to the total variability of foci was assessed by calculating the ratios between intracellular and intercellular CV values for both focus types and subtracting it from one. To this end the average CV values from all fixation time points post exposure were used and noise (N) was calculated according to the formula:

1 $$\begin{aligned} N\: = \left( 1 - \:\frac{{Mean\: intracellular }\: CV { of focus\: frequency}} {{Mean\: intercellular }\: CV { of focus\: frequency}} \right) \times 100\% \end{aligned}$$

For each fixation time point post exposure, the same population of cells was used for both analyses (intracellular CV and intercellular CV), therefore the mean focus frequencies for the averaged intra- and intercellular CV are identical. Only the pairing of cells differs between the two cases. Consequently, differences in averaged CV arise solely from differences in dispersion rather than from differences in mean focus frequencies.

Under this condition, the ratio of CVs simplifies to a ratio of standard deviations, and Eq. (1) can be rewritten as:

2 $$\begin{aligned} N = \left( \frac{\sigma_{\mathrm{intercellular}} - \sigma_{\mathrm{intracellular}}}{\sigma_{\mathrm{intercellular}}} \right) \times 100\%\end{aligned}$$

Consequently, the expression represents the excess dispersion observed in intercellular cell pairs using the intracellular pairs as baseline. We interpret this excess dispersion as variability introduced by noise.

Correlations of foci in two nuclei were measured by calculating Pearson correlation coefficients using Python (version 3.13). Correlation coefficients between foci and nucleus areas were calculated by Pearson Product Moment Correlation using SigmaStat 4.0 (Systat Software Inc., USA).

## Results

**Fig. 2:**
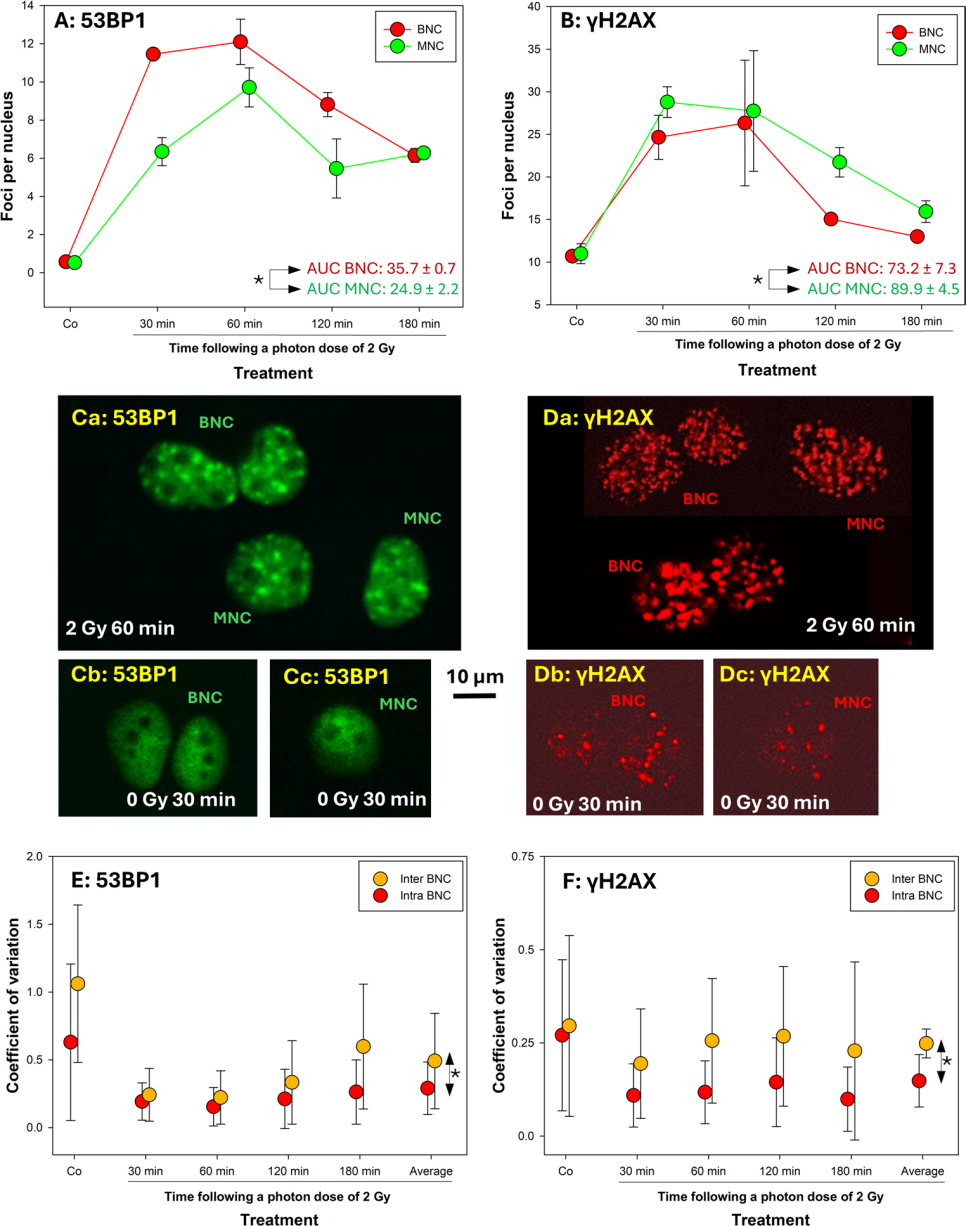
Frequencies of 53BP1 (left panels) and γH2AX (right panels) foci in BNC and MNC. Panels A and B: focus frequencies. Panels Ca, Cb and Cc: exemplary images of 53BP1 foci in BNC and MNC. Panels Da, Db and Dc: exemplary images of γH2AX foci in BNC and MNC. Panel E: Coefficients of variation of focus frequencies calculated for sister nuclei of one BNC (intra BNC) and for nuclei between two BNC (inter BNC). Note that for the sake of clarity the scaling of the Y-axes is different in panel pairs A: B and E: F. Co: control; AUC: area under curve; *: *p* < 0.05

Frequencies of 53BP1 foci in BNC and MNC are shown in Table 1 and in graphical form in Fig. 2A and frequencies of γH2AX foci in BNC and MNC – in Table 1 and in Fig. 2B. Exemplary images of BNC and MNC are shown in Fig. 2C and D. The formation and decay of both focus types followed the typical pattern with the highest frequencies of foci seen during 60 min post exposure. The frequency of 53BP1 foci was significantly higher in nuclei of BNC than of MNC. A reversed, but weaker, result was seen for γH2AX foci. Overall, the frequency of radiation-induced γH2AX foci was 2–3 times as high as that of 53BP1 foci. For control cells, the difference was approximately 10-fold.

**Table 1:** Mean frequencies and standard deviations (std) of 53BP1 and γH2AX focus frequencies in nuclei of binucleated (BNC) and mononucleated (MNC) cells

Focus type	Treatment	Mean BNC	SD BNC	BNC scored	Mean MNC	SD MNC	MNC scored
53BP1	Control	0.58	0.12	251	0.53	0.111	90
53BP1	2 Gy, 30 min	11.45	0.26	221	6.35	0.74	83
53BP1	2 Gy, 60 min	12.1	1.19	235	9.71	1.02	71
53BP1	2 Gy, 120 min	8.81	0.63	194	5.46	1.55	78
53BP1	2 Gy, 180 min	6.15	0.36	138	6.28	0.31	90
γH2AX	Control	10.69	0.27	35	10.99	1.16	70
γH2AX	2 Gy, 30 min	24.67	2.59	65	28.79	1.8	77
γH2AX	2 Gy, 60 min	26.33	7.38	22	27.75	7.08	30
γH2AX	2 Gy, 120 min	15.05	0.51	44	21.74	1.74	85
γH2AX	2 Gy, 180 min	13.00	0.0	22	15.94	1.27	37

Standard deviations (SD) values are from the means of the repeated experiments. Cells scored represent the sums of cells scored in all repeats

Intra BNC and inter BNC CV levels are shown in Fig. 2E for 53BP1 and in Fig. 2F for γH2AX. The CV in control cells was higher for 53BP1 as compared to γH2AX foci. The levels of CV in cells exposed to radiation was similar for both focus types. For both focus types, the average intra BNC CV of foci induced by 2 Gy of photons was significantly lower than the inter BNC CV. In relative terms, the effect was somewhat stronger for γH2AX than for 53BP1 foci.

For radiation-induced foci, the noise calculated according to Eq. 1 was 28.1% for 53BP1 foci and 50.3% for γH2AX foci. For control foci, the noise calculated according to Eq. 1 was 40.6% for 53BP1 foci and 8.5% for γH2AX foci.

**Fig. 3:**
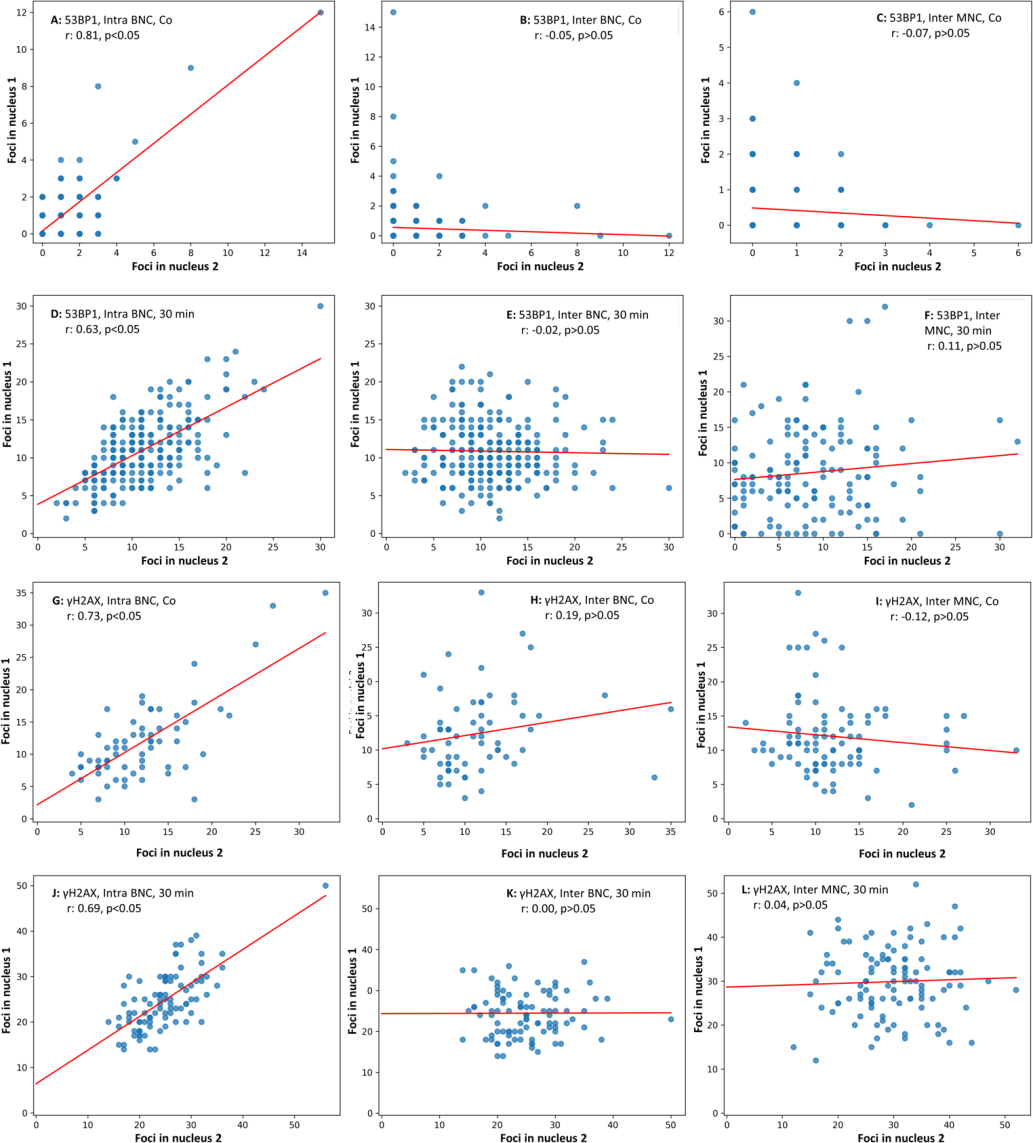
Correlations of 53BP1 (panels A-F) and γH2AX (panels G-L) foci in sister nuclei of BNC (left panels), between nuclei of two BNC (middle panels) and between nuclei of two MNC (right panels). Shown are data for control (A-C and G-I) and 30 min (D-F and J-L) post exposure. Correlations for all time points are given in supplementary figure S1

Correlation analyses between foci in sister nuclei of one BNC, between foci in nuclei of two BNC and between foci in nuclei of two MNC are shown, respectively, in Fig. 3 left, middle and right panels. Panels A, B, C show foci in 53BP1 control cells; panels D, E, F show 53BP1 foci in cells harvested 30 min post 2 Gy; panels G, H, I show γH2AX foci in control cells; panels J, K, L show γH2AX foci in cells harvested 30 min post 2 Gy. Correlation coefficients for all other fixation time points after 2 Gy are shown in supplementary Fig. 1. A significant correlation (see r values) was seen only for control and 2 Gy-induced foci in sister nuclei of BNC (Fig. 3, panels A, D, G, J). The focus numbers between nuclei of two BNC and of two MNC did not show any significant correlation (r values less than 0.19).

**Fig. 4:**
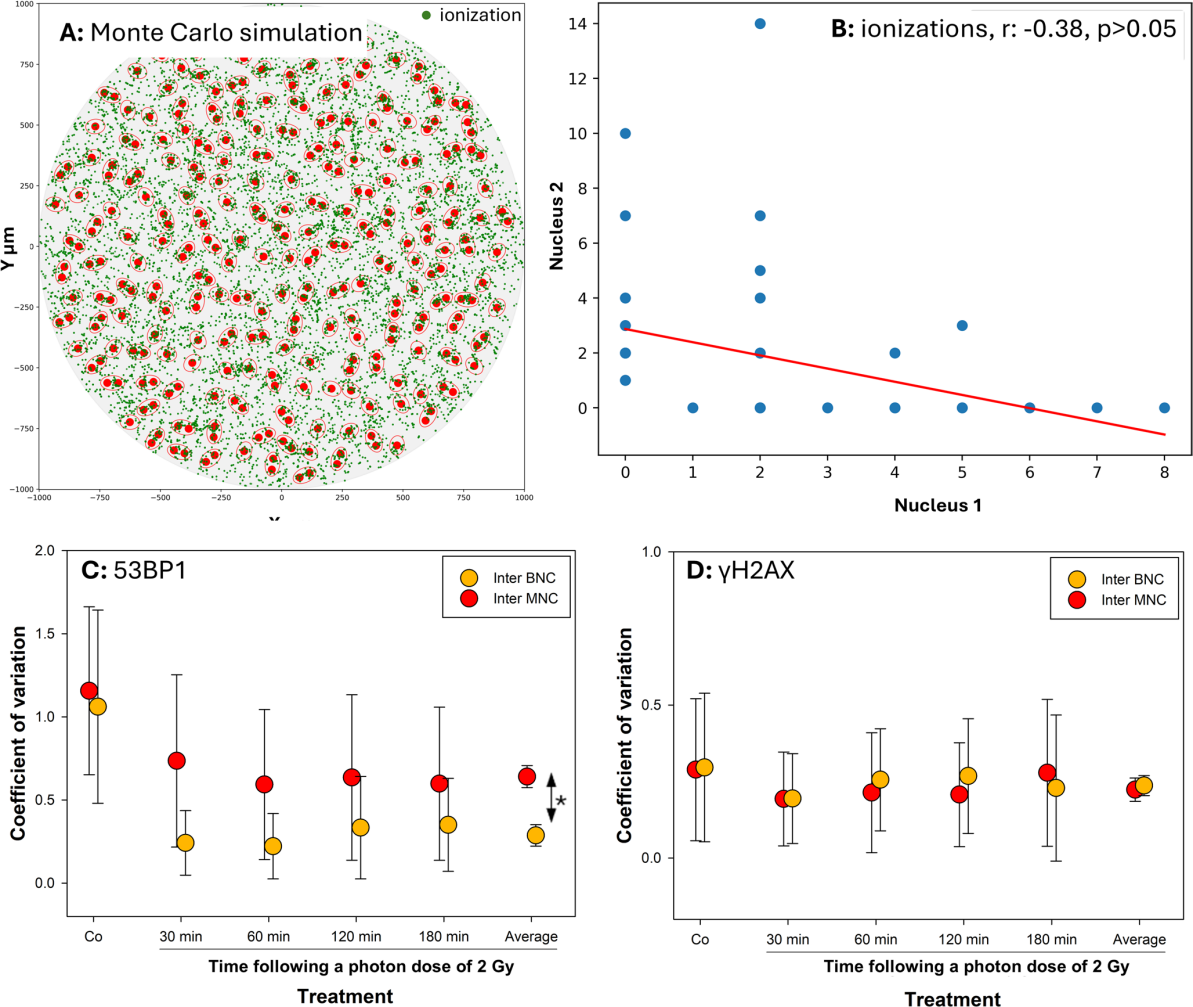
Panels A and B: Geant4-DNA Monte Carlo simulation of ionisations in nuclei of BNC. Panel A shows a visualisation of ionisations in BNC and MNC plated on a 50 μm phantom. Panel B shows a correlation between ionisations in sister nuclei of BNC. Panel C shows coefficients of variation of 53BP1 focus frequencies calculated for nuclei between two BNC (inter BNC) and between nuclei of two MNC (inter MNC). Panel C shows coefficients of variation of γH2AX focus frequencies calculated for nuclei between two BNC (inter BNC) and between nuclei of two MNC (inter MNC). Note that for the sake of clarity the scaling of the Y-axes is different in the panel pair C: D

In order to check that the significant correlation observed for both focus types in sister nuclei of BNC is not due to proximity effects, ionisations in nuclei of cells plated on a 50 μm phantom were simulated using the Geant4-DNA Monte Carlo tool and quantified. The distribution of ionisations in randomly positioned BNC and MNC are visualised in Fig. 4A. No correlation was observed for ionisations in sister nuclei of a BNC (Fig. 4B) demonstrating that the significant correlation between focus types in sister nuclei of BNC is not due to proximity effects.

It was interesting to compare the CV levels of 53BP1 and γH2AX foci between nuclei of two BNC (inter BNC) and between nuclei of two MNC (inter MNC). The results are shown in Fig. 4C and D. A significantly lower CV level was seen for inter BNC variability in 53BP1 foci as compared to inter MNC variability. For γH2AX foci the inter BNC variability was not different from the inter MNC variability.

**Fig. 5:**
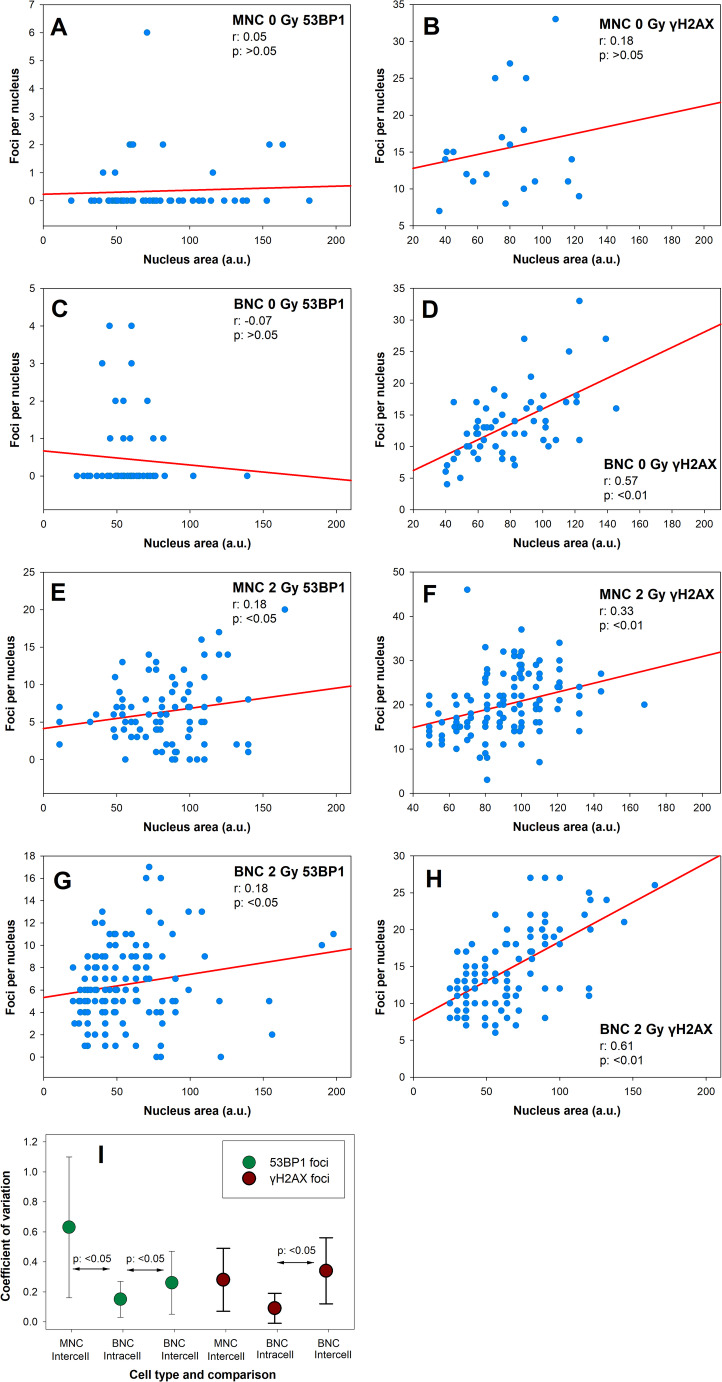
Panels A to H: Correlations between 53BP1 (left panels) and γH2AX (right panels) focus frequencies and areas of cell nuclei given in arbitrary units (a.u.). Results for cells harvested at 180 min p.r. A-B: MNC, 0 Gy; C-D BNC, 0 Gy; E-F: MNC, 2 Gy, G-H: BNC, 2 Gy. Panel I shows coefficients of variation of 53BP1 and γH2AX focus frequencies normalized to the nucleus area calculated for sister nuclei of a BNC (BNC intracell), between single nuclei of two BNC (BNC intercell) and between nuclei of two MNC (MNC intercell)

It was possible that the significant differences in CV values shown in Figs. 2E and F and 4C and D were not due to noise, but to differences in cell cycle distribution of scored nuclei. A way to probe this was to calculate CV values of focus frequencies that were normalised to the nucleus area, assuming that the area of a nucleus reflects its DNA content. To this end, areas of nuclei of BNC and MNC were calculated for images of control cells and cells fixed 180 min post exposure. Foci were scored in the measured nuclei and it was first checked if a relationship exists between the focus frequency and nucleus area. The results shown in Fig. 5A and H confirm that such a relationship exists both for MNC and BNC and it is particularly strong in exposed cells (panels E, F, G, H) and for γH2AX foci (panels F and H). CV values calculated for BNC and MNC showed a significantly lower level of BNC intracell variability as compared to BNC intercell variability and significantly higher intercell variability in MNC as compared to BNC, but only for 53BP1 foci (Fig. 5I).

## Discussion

The aim of the investigation was to test the hypothesis that the intrinsic in vitro radiation sensitivity of individual cells of the same cell line varies and that the variability, that can be referred to as noise, is independent of the stochastic nature of energy deposition in nuclei. The hypothesis was tested by comparing 53BP1 and γH2AX focus frequencies between sister nuclei of BNC and between single nuclei of two BNC. The results demonstrated that, for both focus types, the frequency of foci in sister nuclei of a BNC are correlated and show a level of noise that is lower than the noise between single nuclei of randomly selected BNC. Assuming that both nuclei of a BNC show the same momentary intrinsic radiosensitivity, this result means that the difference in focus frequency observed between sister nuclei results solely from random energy deposition, while the difference in focus frequency between nuclei of two randomly selected BNC result from the random energy deposition and from noise. This allowed us to calculate the contribution of noise to the total variability and it was 28.5% for 53BP1 foci and 50.3% for γH2AX foci. The possible reason for this difference is discussed further down.

It is now well understood that even genetically identical cells can demonstrate variable phenotypes and that one source of this variability are random fluctuations in the expression of individual genes (Raj and van Oudenaarden 2008). The fact that genes are expressed in bursts (Gatlin et al. 2025) and that many mRNA molecules are present in low numbers means that these fluctuations do not simply average away but can lead to clearly detectable differences between otherwise identical cells (Eberwine and Kim 2015). The cell-to-cell variability can be seen as the outcome of redundancy in regulatory networks or as a temporal snapshot of asynchronous dynamic processes. It is not clear whether it is a purely cell-autonomous process or if it is driven by communication with other cells or its microenvironment. Gatlin et al. demonstrated that single-cell gene expression variability is intrinsically linked to the function of genes and is primarily driven by highly variable genes (Gatlin et al. 2025). Whatever the mechanism, Dueck et al. suggested that the variability may provide a fitness advantage by enabling a population to adapt to changing environments or to perform diverse tasks (Dueck et al. 2016). In fluctuating, unpredictable environments, a population may benefit by maintaining a diversity of cell phenotypes, each advantageous in a distinct context. Maintenance of a standing diversity may be preferred when a rapid response of at least a subpopulation is advantageous and there is insufficient time for signal transduction (Dueck et al. 2016; Eberwine and Kim 2015). This strategy is referred to as bet hedging (Dueck et al. 2016) and it can be assumed that differences in response of cells to radiation-induced DNA damage result not only from random variation in energy deposition but also from the random variability in the copy number of proteins involved in DDR. On top of this, reaction of cells to stress is influenced by external factors such as temperature (Plodowska et al. 2022). Taken together, the variability in response of cells to DNA damage means that there is no simple degree of intrinsic radiosensitivity that characterizes otherwise identical cells.

The contribution of noise to the total variability was higher for 53BP1 foci than for γH2AX foci. At present it is not possible to say if this difference is due to factors related to scoring of foci or whether it has any specific biological meaning. The frequency of γH2AX foci in BNC and MNC was between two-, and threefold higher than that of 53BP1 foci. It is known that 53BP1 acts downstream of γH2AX: RNF8 promotes MDC1-dependent signalling, and RNF8/RNF168-mediated ubiquitination facilitates 53BP1 accumulation at the DNA double strand break (DSB) site (Mailand et al. 2007; Bekker-Jensen and Mailand 2010). In general, when detected with monoclonal antibodies, γH2AX and 53BP1 show a similar frequency and they colocalise (Jezkova et al. 2018). However, several studies report that BRCA1–CtIP–mediated resection can antagonize 53BP1 recruitment even when γH2AX is present (Escribano-Diaz et al. 2013). Similarly, defects in the RNF8–RNF168 ubiquitin cascade can prevent 53BP1 binding despite H2AX phosphorylation (Mailand et al. 2007) or loss of upstream factors such as MDC1 can block 53BP1 (Stucki et al. 2005). It is also possible that the difference seen by us is due to the different detection techniques applied to visualize both focus types. The U2OS cells used in this study are transfected with a plasmid coding for the 53BP1 protein tagged by green fluorescent protein (GFP) (Bekker-Jensen et al. 2005). This allows visualisation of foci without the use of monoclonal antibodies. It can be expected that 53BP1-GFP proteins compete with native 53BP1 proteins in the formation of foci, probably resulting in weak fluorescence of foci and, consequently, reduced signal-to-noise ratio and low detectability. γH2AX foci were detected with monoclonal antibodies leading to strong signals and high numbers of detected foci. Based on this it could be argued that the noise of γH2AX foci is more representative for the actual level of noise in the cellular DDR of U2OS cells. On the other hand, it is known that the noise is gene-specific (Gatlin et al. 2025) and we do not know how far this noise specificity applies to *53BP1* and *ATM* and other DNA damage repair genes.

An observation that is worth highlighting in light of the above discussion is that the mean coefficient of variation for radiation-induced 53BP1 foci was higher in BNC as compared to MNC, while this was not the case for γH2AX foci. This result may be associated with the generally higher frequencies of 53BP1 foci observed in BNC as compared to MNC, while the reversed result, although at lower degree, was obtained for γH2AX foci. As discussed above, the 53BP1 focus levels depend on the number of available proteins that can be recruited to the DSB site and results of investigations on protein expression in MNC/euploid cells vs. BNC/polyploid cells suggest generally higher copy numbers in BNC/polyploid cells (Windmueller et al. 2020; Kreutz et al. 2017; Yahya et al. 2022; Dong et al. 2024). Hence, it can be assumed that the higher level of 53BP1 focus frequencies in BNC as compared to MNC results from higher copy numbers in the former.

Another possible explanation for the higher level of 53BP1 focus frequencies in BNC as compared to MNC is that the analysed BNC and MNC cells were in different phases of the cell cycle. Cytochalasin B modulates the polymerization of actin microfilaments retarding cell cycle progression and differentiation (Pampanella et al. 2023). Thus, it is possible that in our setup many BNC accumulated in the G_1_ phase of the cell cycle, while MNC were more equally distributed over all cell cycle phases. 53BP1 activity/recruitment is maximal in G_1_ of the cell cycle and is actively antagonized in S/G_2_ where BRCA1-mediated resection and homologous recombination repair dominates (Escribano-Diaz et al. 2013; Mirza-Aghazadeh-Attari et al. 2019). In contrast, the phosphorylation of the H2AX histone protein is more independent of the cell cycle phase (Podhorecka et al. 2010; Valente et al. 2022). It is possible that both mechanisms discussed above contributed to the higher level of 53BP1 focus frequencies observed in BNC as compared to MNC nuclei. We do not have a plausible explanation for the reversed difference in γH2AX focus frequencies between BNC and MNC. U2OS cells express a constitutively high level of the ATM protein (Gately et al. 1998) and there is no reason to assume that its level or activity is higher in MNC as compared to BNC. Moreover, the difference is largely driven by measurement carried out at 120 min post exposure so that most likely is due to chance. Summing up, the higher mean coefficient of variation for radiation-induced 53BP1 foci in BNC as compared to MNC may be related to the higher focus frequencies in BNC, but the biological meaning of the different variability is not clear and requires further investigations.

A possible confound for the interpretation of variability results is related to possible differences in cell cycle distribution of sister nuclei of BNC vs. nuclei of two BNC. It is known that the frequency of DNA repair foci induced by ionising radiation is directly proportional to the DNA content of the cell nucleus (Lobrich et al. 2010; Kataoka et al. 2006) and it could be argued that the lower intracell focus variability as compared to intercell variability in BNC resulted from a higher degree of synchrony in cell cycle progression of nuclei of the former. A high diversity in the position of the cell nucleus in the cell cycle would result in a high level of focus variability. We checked this possibility by calculating CV of focus frequencies normalised to the nucleus area. The nucleus area on microscopic slides was used as an indicator of the DNA content (Rassamegevanon et al. 2018) and we could confirm a direct relationship between radiation-induced focus frequency and the nucleus area. By normalising the focus frequency to nucleus area, the confounding effect of differences in cell cycle position was eliminated. CV values calculated for the normalised focus frequencies in BNC showed a significantly lower intracell variability as compared to intercell variability demonstrating that the noise observed by us is not a consequence of diversity in cell cycle distribution.

Our study has several limitations that should be addressed. Because the initial plan included the attempt to analyse repair foci in micronuclei, cells were irradiated with 2 Gy before addition of cytochalasin B to induce micronuclei. However, too few scorable micronuclei were for a meaningful analysis, so the plan was not pursuit. It cannot be ruled out that the first irradiation induced some form of adaptation mechanism that modulated the response to the second radiation dose. Adaptive responses have been shown to be induced by radiation doses below 100 mGy and data on adaptive responses exist for doses in this range (Tapio and Jacob 2007; Grdina et al. 2013). Consequently, it is not clear how far the first dose of 2 Gy influenced the response of cells to the second dose. However, there is no reason to assume that it differentially influenced the observed intra-, and intercell variation of focus frequencies because all cells were treated in the same way.

Different detection techniques of 53BP1 and γH2AX foci were applied. We used the 53BP1-GFP-transfected U2OS cells to detect 53BP1 foci because they do not require using histochemistry and they were successfully used in several of our previous studies (Sollazzo et al. 2018; Plodowska et al. 2022, 2025). Due to the relatively low signal to nose ratio of the 53BP1-GFP foci (see Fig. 2, panels Ca, Cb and Cc), we did not counterstain the nuclei and, for the sake of consistency, the same attitude was applied to γH2AX foci analysis. Lack of counterstaining prevented the possibility of measuring the DNA content of nuclei by image analysis techniques and we used the crude approach of measuring the X and Y diameters of the nuclei by a ruler. Moreover, we analysed foci in one Z-plane only instead of going for a Z-stack analysis through the nucleus to detect all foci within. Finally, we used a single for reasons described in the introduction.

The consequence of the approaches is that the reported results may not provide absolute quantitative information regarding the mechanisms of DNA damage response to radiation. Also, we do not know how far the results are reproducible in a normal cell line. The objective of the present study was not to determine the absolute number of DNA double-strand breaks per nucleus and study basic mechanisms of DDR, but rather to compare the relative distribution of foci frequencies between neighbouring nuclei acquired under identical experimental and imaging conditions. Importantly, all nuclei included in each comparison originated from the same cell line and treatment condition and were imaged in the same focal plane using identical acquisition settings. We feel that under these conditions, no systematic bias was introduced limiting the relative comparisons that constitute the basis of our analysis. Nevertheless, in view of the limitations we wish to stress that the study represents a proof of principal method approach that we wish to share with the research community to tackle this question using more rigorous approaches.

## Supplementary Information

Below is the link to the electronic supplementary material.

Supplementary Material 1

## Data Availability

No datasets were generated or analysed during the current study.
