# Supplementary material for: Assessing the variation of DNA damage response of individual human cells exposed to photon radiation
Source: Radiat Environ Biophys. 2026 Jun 16;65(3):943–55. doi: 10.1007/s00411-026-01232-9 (PMC13486072; doi:10.1007/s00411-026-01232-9)

Supplementary Figure 1A: Correlations of 53BP1 foci in sister nuclei of BNC (left panels), between nuclei of two BNC (middle panels) and between nuclei of two MNC (right panels).

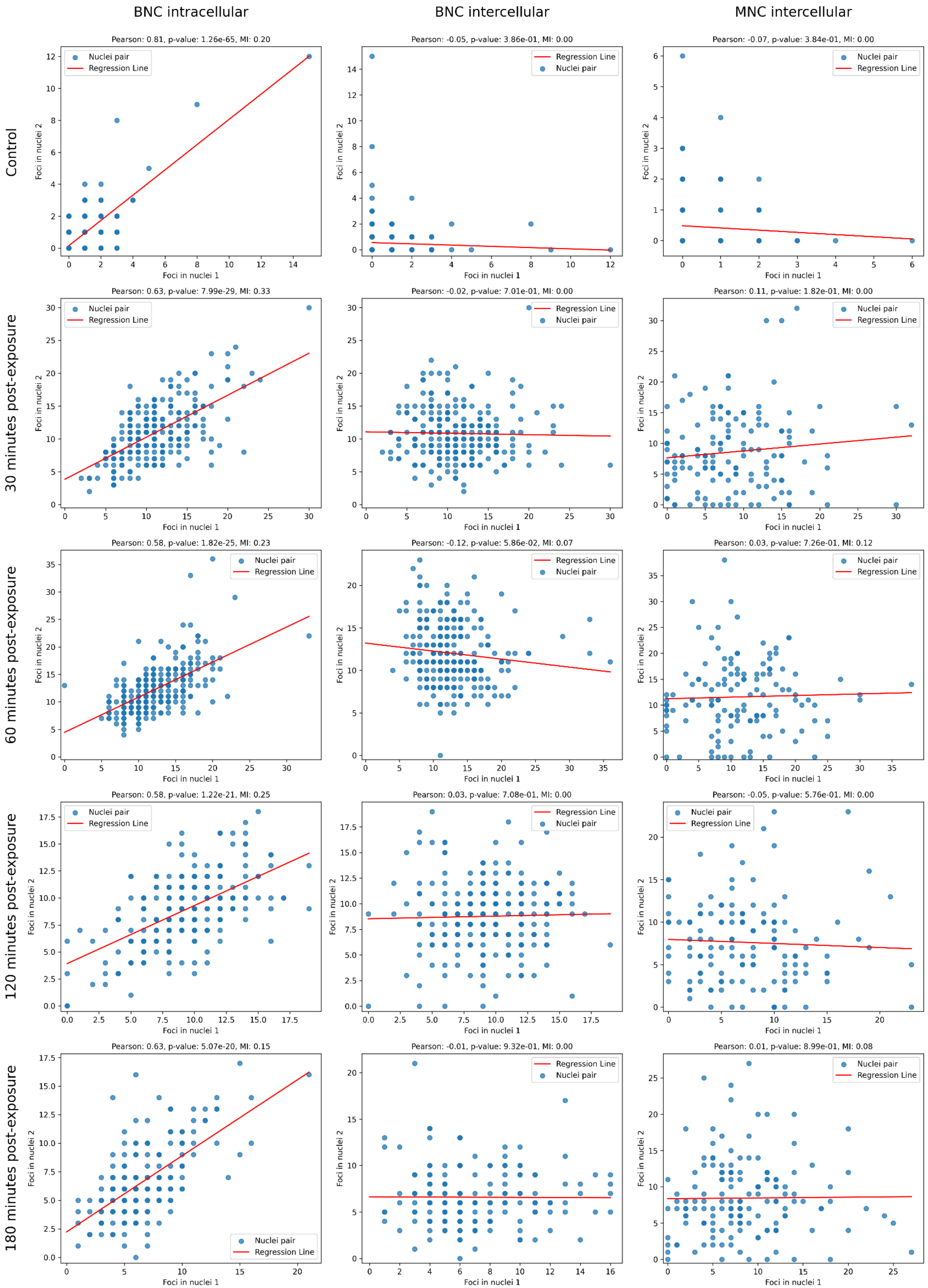

Supplementary Figure 1B: Correlations of γH2AX foci in sister nuclei of BNC (left panels), between nuclei of two BNC (middle panels) and between nuclei of two MNC (right panels).

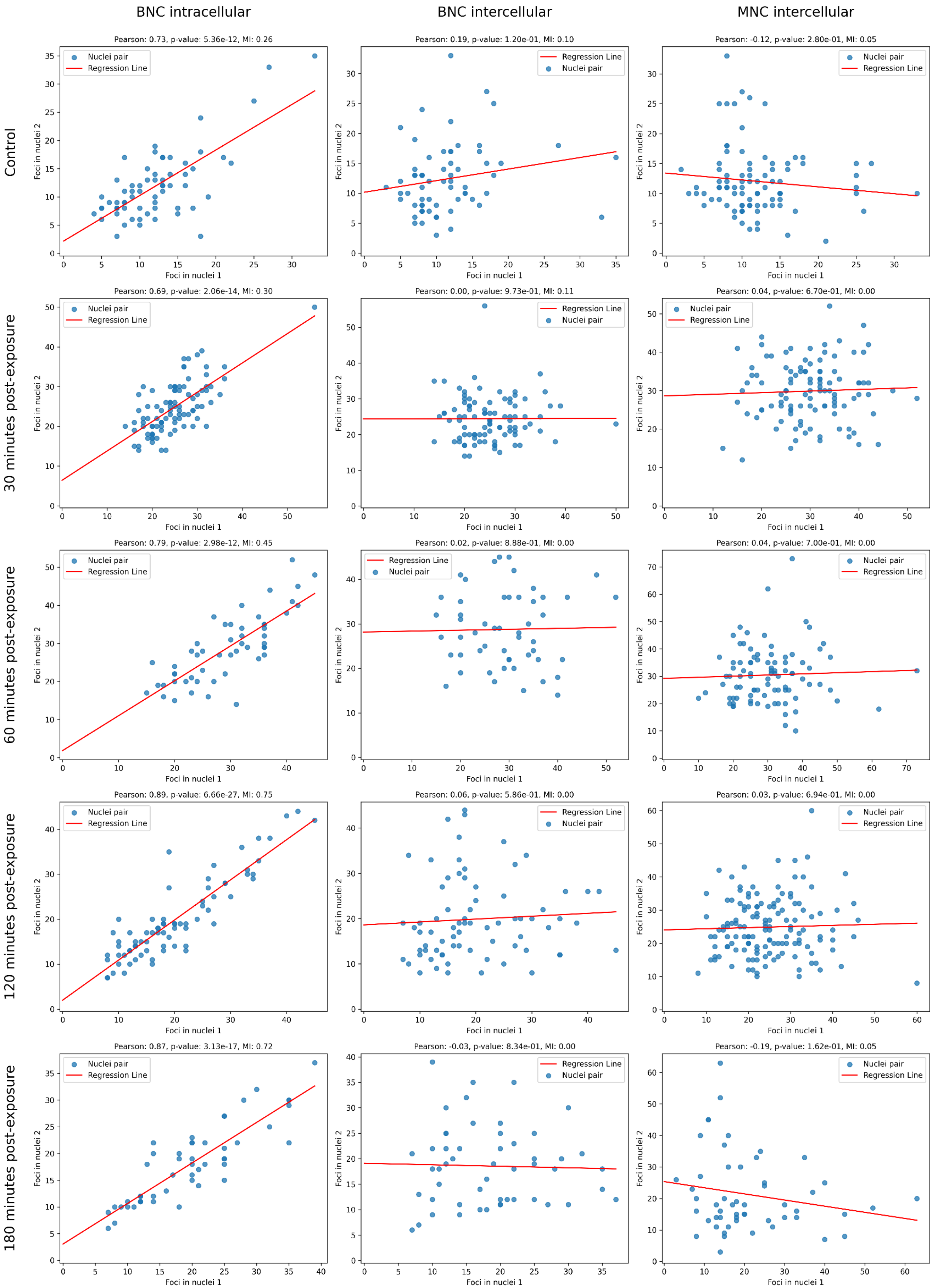

Supplement: Supplementary file 1 [file 411_2026_1232_MOESM1_ESM.pdf]
